# Estimating the plasma volume by infusing albumin: a retrospective feasibility study

**DOI:** 10.1186/s40635-025-00743-x

**Published:** 2025-03-20

**Authors:** Robert G. Hahn, Joachim H. Zdolsek

**Affiliations:** 1 Anesthesia and Intensive Care, Karolinska Institutet at Danderyds Hospital (KIDS), 152 86 Danderyd, Stockholm, Sweden; 2 Department of Biomedical and Clinical Sciences (BKV), Linköping University, Linköping, Sweden

**Keywords:** Plasma volume, Plasma albumin, Pharmacokinetics, Anthropometry, Body composition

## Abstract

**Background:**

The combined changes in plasma albumin and blood hemoglobin can probably be used to estimate the plasma volume (PV) when albumin is infused. However, the optimal setup, timing of the blood sampling, and the importance of capillary leakage to the calculations are unclear.

**Methods:**

In this technical vignette, we estimated the PV using retrospective data on plasma albumin and blood hemoglobin obtained during intravenous infusion of 3 mL/kg of 20% albumin over 30 min in 41 volunteers and 45 patients. We used a manual and a kinetic correction for capillary leakage of albumin. The results were compared to the mean of two anthropometric equations derived via tracer methods.

**Results:**

The anthropometric PV was 3.00 ± 0.63 L (mean ± SD). The strongest linearity between the albumin-derived and anthropometric PV was obtained at the end, and 10 min after the end, of the 30-min infusions; the correlation coefficient was 0.75 over this time frame. The difference between the two measures (the prediction error) was 0.31 ± 0.56 L but the SD was only half as high for PVs< 2.5 L than for larger PVs. There was slightly stronger linearity and better accuracy, but no better precision, when data were corrected for capillary leakage.

**Conclusion:**

This study suggests how an evaluation of this method using isotopes can be conducted. Changes in plasma albumin and blood hemoglobin have the best chance to accurately indicate the PV at the end of, or 10 min after, a 30-min infusion of albumin. Subtraction of 0.3 L from the PV is sufficient to correct for capillary leakage of albumin.

**Supplementary Information:**

The online version contains supplementary material available at 10.1186/s40635-025-00743-x.

## Introduction

Hemodynamic measurements are widely used aids in clinical decision-making, while measurements of body fluid volumes are more difficult to apply bedside due to the required laboratory techniques [1]. However, quantitation of the plasma volume (PV) would be of interest in critical and uncertain clinical situations in sepsis, trauma, burns, hemodialysis, and hypovolemic episodes during lengthy surgeries. An ideal approach would be to estimate the PV as part of a therapeutic intervention that is performed for another purpose. One such possibility exists when exogenous albumin is used for PV support. Clinicians may prefer albumin for PV expansion instead of crystalloids due to the long-lasting effect [2].

The possibility of estimating the PV by administering albumin was explored by Margarson and Soni 20 years ago [3–5]. They infused 200 mL of 20% albumin over 90 s into a central vein and assumed that the change in plasma albumin at 1 min would be proportional to the PV before albumin was given. Measuring plasma albumin so soon after the injection makes it possible to overlook the confounding effect of capillary leakage of albumin. Nevertheless, their approach has not gained widespread acceptance.

In the present study, we aimed to re-assess the practical feasibility of measuring the PV in this way but changed Margarson and Soni’s setup to make the measurement more clinically applicable. First, we extended the infusion time to 30 min as we believe that 90 s might cause adverse effects. Second, we accounted for the PV expansion induced by the exogenous albumin by correcting plasma albumin for hemodilution. Third, the administered amount of albumin was corrected by the hematocrit factor of 0.91, because approximately 10% of exogenous albumin participates in an extravascular rapid-exchange circuit that is probably located in the liver sinusoids [6]. Fourth, we infused fluid and sampled blood in peripheral veins and, finally, we quantified the impact of capillary leakage of albumin on the calculations. We calculated PVs during 86 infusion experiments in this way and compared the results to PVs derived by two widely used anthropometric methods that both rely on tracer techniques (Evan’s blue dye and radioactive iodine tagged to albumin molecules) [7, 8]. The result could serve as the basis for how an evaluation of this method using isotopes should be conducted. The hypothesis was that the albumin-derived PV and the anthropometric PV correlate well and that a point in time can be identified when their agreement is strongest.

## Methods

### Subjects

This present report is a secondary publication to four publications in which 3 mL/kg of 20% albumin (approximately 200 mL) was administered to 74 subjects over 30 min at a constant rate. Moreover, 12 volunteers received the same amount of albumin but as a 5% albumin solution over 30 min. We chose to administer 200 mL of 20% albumin because it is a commonly infused volume when albumin treatment is clinically indicated. PV expansion of 20% (≈400 mL) is also desired when fluid kinetics is based on plasma dilution. Smaller volumes would increase the confounding effects of measurement errors on the calculations.

The current study groups included 41 healthy volunteers, 30 patients with inflammation (following surgery or burns) and 15 patients studied during lengthy surgery that involved minor trauma and negligible blood loss, such as correction of pro-and retrognathia, breast reconstruction after ablation mammae, and removal of cholesteatoma.

The group with inflammation included two cohorts. The first cohort included 15 patients who were studied in the first morning after major surgery that had lasted for 5.9 h (mean) and involved an average perioperative blood loss of 700 mL. These patients were judged to be normovolemic at the time of the experimental infusion.

The second cohort was the same size as the first one. These patients were studied 3–14 (mean 7) days after burn injuries that covered >6% (median 15%) of the total body surface area; these burns were still severe enough to require hospital care. Both cohorts had plasma concentrations of C-reactive protein averaging approximately 80 mg/L and were then considered to have inflammation of intermediate intensity. By contrast, the C-reactive protein level was at baseline or close to the baseline of 5 mg/L in the volunteers and in the patients undergoing surgery.

Details from these experiments have been published in *Anesth. Analg.* **2019**, *129,* 1232–1239; *Anesth. Analg.* **2022,** *134,* 1270–1279; *Crit. Care* **2024,** 24, 191; *Acta Anaesthesiol. Scand.* **2022**, *66,* 847–858.

All experiments were performed in accordance with the Declaration of Helsinki. Each subject provided written informed consent to participate before any experiments were initiated. Reporting adhered to the STROBE checklist.

### Data collection

Blood (10 mL) was withdrawn over 15 exactly timed periods over 5 h after starting the infusion (0, 10, 20, 30, 40, 50, 60, 75, 90, 120, 150, 180, 210, 240 and 300 min). Whole blood was analyzed for hemoglobin (B–Hb) concentration and the hematocrit (Hct). Plasma was collected for the plasma albumin concentration (P-Alb). The hospital’s certified laboratory used clinical chemistry analyzers for these measurements. The coefficient of variation was 0.7% for B–Hb and 2.8% for P-Alb based on duplicate samples obtained at baseline in 34 of the experiments.

### Anthropometry

The blood volume (BV) in all subjects was estimated according to the equations based on sex, height (H), and body weight (BW) published by Nadler et al*.* [7] and Retzlaff et al*.* [8].

Nadler et al*.* calculated BV in 155 men and women using albumin molecules tagged with radioactive iodine. Height was expressed in meters and BV was in liters:

$$\begin{gathered} {\text{BV}} = 0.3669\,{\text{H}}^{3} + 0.03219\,{\text{BW}} + 0.6041\,\left( {{\text{males}}} \right) \hfill \\ {\text{BV}} = 0.3561\,{\text{H}}^{3} + 0.03308\,{\text{BW}} + 0.1833\,\left( {{\text{females}}} \right) \hfill \\ \end{gathered}$$

Retzlaff et al*.* recruited 78 volunteers and measured the red cell volume with chromium-tagged erythrocytes and PV with albumin marked with Evan’s blue. Height was expressed in centimeters and BV in milliliters:

1 $${\text{BV}} = 31.9\,{\text{H}} + 26.3\,{\text{BW}}{-}2402\left( {{\text{males}}} \right)$$

2 $${\text{BV}} = 56.9\,{\text{H}} + 14.1\,{\text{BW}}{-}6460\left( {{\text{females}}} \right)$$

The BV was converted to PV by multiplication with (1—hematocrit).

### Calculation of plasma volume (PV)

The PV at baseline can be estimated from the change in Hb and P-Alb during infusion of exogenous albumin. The equations are:

3 $${\text{PV}} = 0.91*{\text{Infused}}\,{\text{albumin}}\,{\text{mass}}/\left[ {{\text{P-Alb}_t}\left( {1 + {\text{DIL}}} \right){-}{\text{P-Alb}_0}} \right]$$

4 $${\text{DIL}} = [(({\text{B-Hb}_0} - {\text{B-Hb}_t})/{\text{B-Hb}_t}) - 1]/(1 - {\text{Hct}}_{0} )$$

where the index 0 denotes the baseline and the index *t* a later time. The factor 0.91 is the f-cell ratio introduced, because 10% of the albumin is quickly translocated to a space just outside the circulating blood (<1 min) [5]. No correction for sampled blood volume (140 mL during 5 h) was applied.

The PV equation (Eq. 3) is a development of a pharmacokinetic “dilution principle” which holds that volume of distribution equals the dose divided by the plasma concentration. However, the difference (P-Alb_t_—P-Alb_o_) is applied here, because P-Alb has a starting concentration. Moreover, a correction of P-Alb_t_ for DIL is used, because the PV expansion dilutes P-Alb and then gives it a falsely low value. Hence, [P-Alb_t_ (1 + DIL)] gives the P-Alb that would result if no PV expansion occurred. Using this mathematical correction, the equation yields the baseline PV, while the expanded PV is obtained when correction for DIL is *not* performed.

DIL is the fractional plasma dilution as given by the changes in B–Hb; this equation yields a linear relationship between dilution and volume expansion (hemodilution equations are usually written with the baseline Hb in the nominator but then provide exponential relationships). The DIL equation is mathematically derived in the Supplemental Data File 1 of Anesth Analg 2022; 134: 1270–1279.

### Corrections for capillary leakage

The PV was calculated at 11 intervals of 10–15 min up to 180 min. Three ways were used that only differed depending on how albumin capillary leakage was handled:

1. **No correction** of capillary leakage.
2. Correction based on the elimination rate constant (*k* value) obtained by **manual calculation** that included the terminal 2–3 h of the 5-h experiments. Specifically, the change in DIL-corrected ΔP-alb is log-transformed and placed in the *y*-axis when a clear decrease of this product over time is detected. The specific time is placed on the *x*-axis, and the *k* value is the slope of the regression between the two variables. The used equation is shown below.
3. **Kinetic correction**. A one-compartment kinetic model was built to analyze the DIL-corrected ΔP-Alb over the entire experiment. Here, the *k* value equals the difference between the capillary leakage of albumin and the lymphatic return of albumin. This *k* value always has a positive value during equilibration of an administrated surplus of albumin. The involved mathematical calculations have been described in detail elsewhere [9].

In Corrections 2 and 3, the infused albumin mass was corrected for the *k* value as follows:

5 $${\text{Remaining}}\;{\text{albumin}} = 0.91*{\text{Infused}}\;{\text{albumin}}\;{\text{mass}}*\left( {{\text{e}}^{{{-}kt}} } \right)$$

where *t* is the time in minutes from the initiation of the infusion. This is the basic equation for mono-exponential decay that governs nearly all biological processes.

### Statistics

The results were presented as the mean ± standard deviation (SD). Correlations between the albumin-based and anthropometric PV were studied by linear regression analysis, where r is the correlation coefficient. We also report the coefficient of determination, *r*^2^, which is the proportion of *y* that can be explained by *x*. The prediction error was obtained as the difference between the albumin-based and the anthropometric PV, and individual differences between these variables were illustrated by Bland–Altman plots. Patient groups were compared using one-way ANOVA followed by the Scheffé test.

## Results

### Basic data

Demographic, baseline data, and selected results are shown in Table 1.

**Table 1:** Demographic data and baseline characteristics

	Healthy volunteers (1)	Inflammation (2)	Surgery (3)	Statistics
Number of participants	41	30	15	
Age (years)	29 ± 11	55 ± 17	46 ± 16	0.001, 1 < 2, 3
Sex (% women)	46%	53%	67%	0.40
Length (cm)	174 ± 12	173 ± 11	172 ± 8	0.73
Body weight (kg)	74 ± 12	84 ± 19	71 ± 16	0.013; 2 > 1, 3
MAP (mmHg)	92 ± 9	80 ± 12	72 ± 12	0.001; 1 > 2, 3
P-creatinine (µmol/L)	81 ± 16	73 ± 16	76 ± 14	0.09
P-platelets (10^9^/L)	219 ± 55	399 ± 163	203 ± 36	0.001; 2 < 1, 3
B–Hb (g/L), baseline	136 ± 10	105 ± 16	128 ± 10	0.001; 2 < 1, 3
Hematocrit, baseline	0.40 ± 0.03	0.32 ± 0.05	0.40 ± 0.01	0.001; 2 < 1, 3
P-Alb (g/L), baseline	40 ± 3	24 ± 4	37 ± 2	0.001; 2 < 1, 3
P-Alb (g/L), 30–40 min	45 ± 3	33 ± 4	43 ± 3	0.001; 2 < 1, 3
P-dilution, 30–40 min	0.15 ± 0.07	0.15 ± 0.05	0.13 ± 0.06	0.20
Anthropometric PV (L)	2.85 ± 0.51	3.36 ± 0.68	2.68 ± 0.48	0.002; 2 > 1, 3
*k* manual (10^–4^ min^−1^)	16.5 ± 8.0	12.5 ± 6.7	8.6 ± 3.5	0.001; 1 < 2, 3
*k* kinetic (10^–4^ min^−1^)	5.0 ± 3.8	7.8 ± 2.7	6.5 ± 3.9	0.005; 1 < 2
Prediction error, 30–40 min (L)				
No correction	0.31 ± 0.51	0.16 ± 0.59	0.59 ± 0.51	0.046; 2 < 3
Manual correction	0.04 ± 0.51	-0.04 ± 0.55	0.19 ± 0.40	0.39
Kinetic correction	0.16 ± 0.54	0.05 ± 0.57	0.16 ± 0.59	0.60
References	[7, 8]	[2, 8]	[9]	

Data are the mean ± SD. Statistical analysis was performed with one-way ANOVA followed by the Scheffé test except “Sex” which was evaluated by contingency table analysis. *MAP *mean arterial pressure

### Anthropometry

The blood volume (BV) at baseline was derived from the anthropometric equations which were converted to plasma volume (PV) by applying the measured hematocrit. Nadler et al*.* reported a PV of 3.02 ± 0.62 L and Retzlaff et al. reported 2.98 ± 0.64 L. The mean value of the two calculations was 3.00 ± 0.63 L, which was used for further comparisons.

### Linearity

**Fig. 1:**
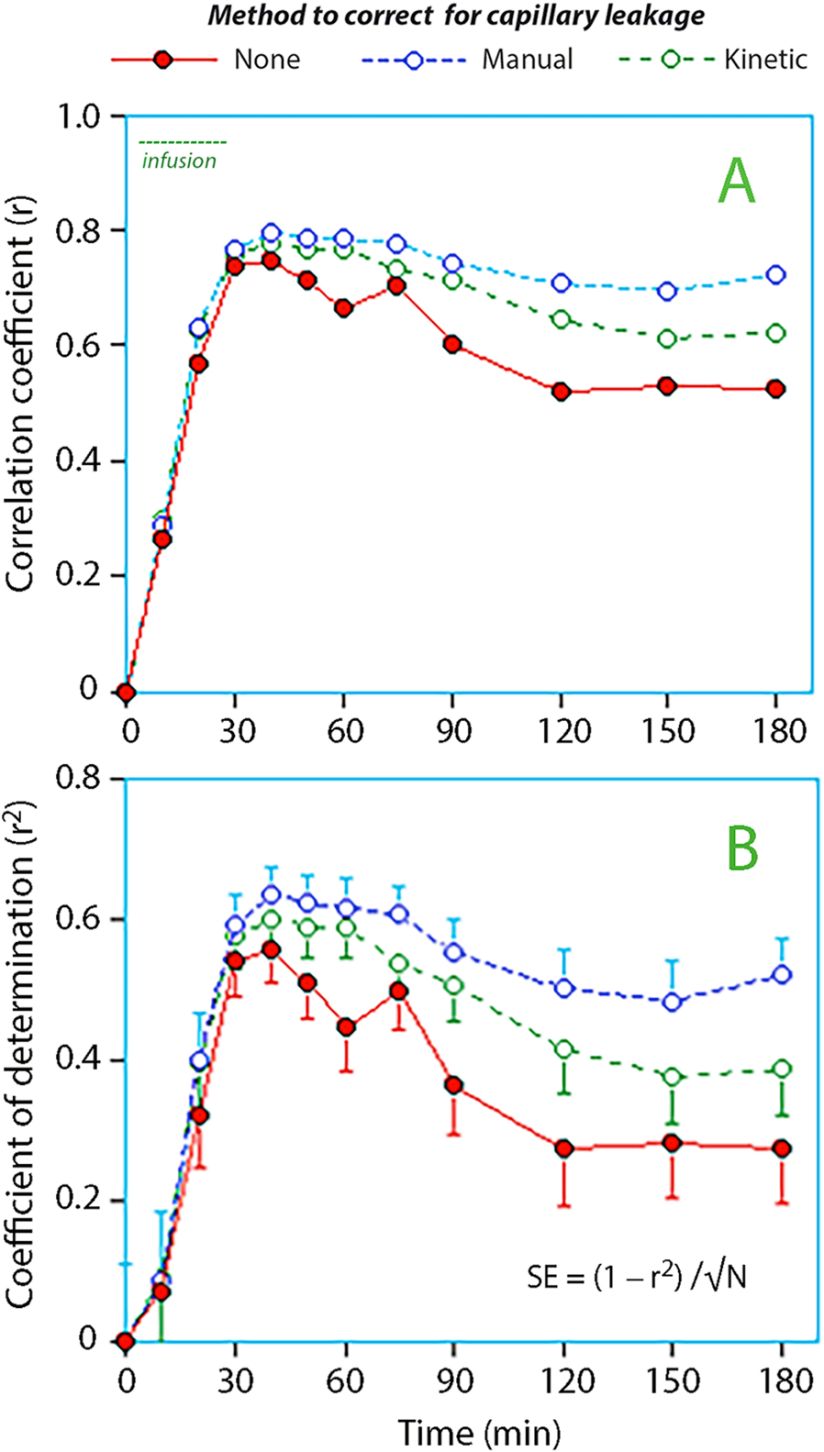
Linearity for the relationship, expressed as **A** the correlation coefficient and **B** coefficient of determination, between the plasma volume as obtained from the mean of two anthropometric equations (by Nadler et al*.* and Retlaff et al*.*) and the plasma volume calculated from blood hemoglobin and plasma albumin measurements made during and at various times during and after a 30-min infusion of albumin. The calculations could disregard the capillary leakage of albumin or include it as derived by a manual or a kinetic method. SE = standard error

The coefficient of determination (*r*^2^) was calculated for the relationship between the anthropometric PV and the albumin-based PV. Figure 1 shows *r*^2^ over time for the three approaches to calculate the albumin-based PV.

This plot demonstrates that the strongest linearity (highest *r*^2^) was obtained at 30 and 40 min, i.e., at the end of the infusion and during the subsequent 10 min. The linearity then became progressively worse.

**Fig. 2:**
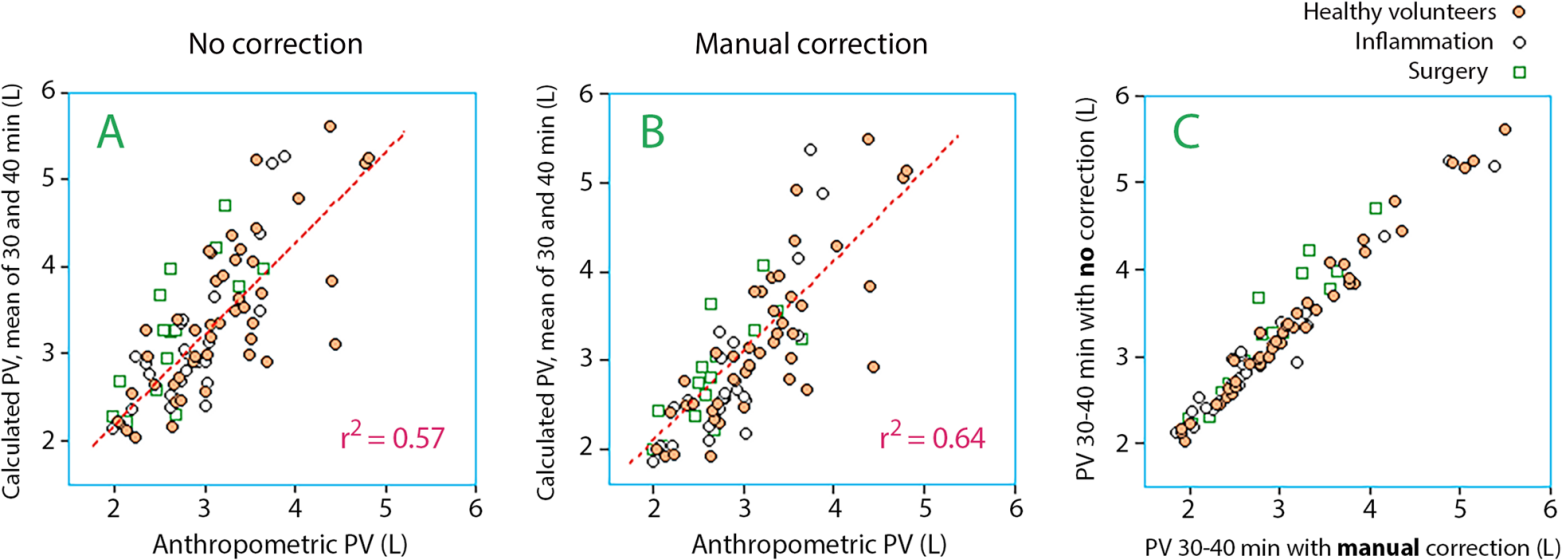
Relationship between the plasma volume (PV) as obtained from the mean of two anthropometric equations and the mean PV based on blood hemoglobin and plasma albumin measurements performed at 30 and 40 min of 30-min intravenous infusion of albumin. The calculations could disregard the capillary leakage of albumin (**A**) or include it as derived by a manual method (**B**). The kinetic method (not shown) yielded a relationship that was quite similar to subplot B. Each point is one infusion experiment

Using the mean value of these two timepoints slightly strengthened the linearity. The correlation coefficient 0.75 when no correction for capillary leakage was applied (Fig. 2A), 0.80 when the terminal half-life was calculated manually (Fig. 2B), and 0.79 when the half-life was taken from the kinetic analysis of the entire curve. Capillary leakage affected the PV only modestly at 30–40 min (Fig. 2C).

The linearity did not change when the 12 experiments with 5% albumin (instead of 20%) were disregarded. Moreover, the linearity did not improve by adding the measurements taken at 50 min to those from 30 to 40 min.

### Prediction error

**Fig. 3:**
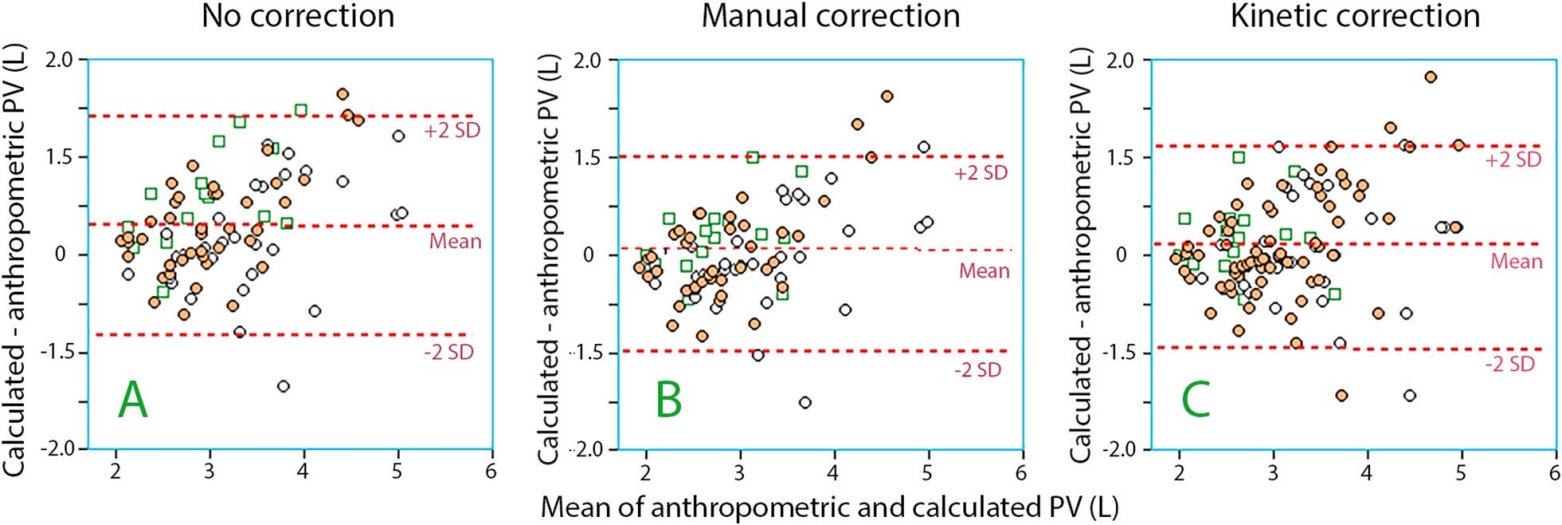
Bland–Altman plots showing the mean of the anthropometric and calculated plasma volume (PV, based on changes in B–Hb and plasma albumin) versus the difference between these two volumes depending on the mode of correcting PV for capillary leakage of albumin. Each point is one infusion experiment and the color indications the same as in Fig. 2

**Fig. 4:**
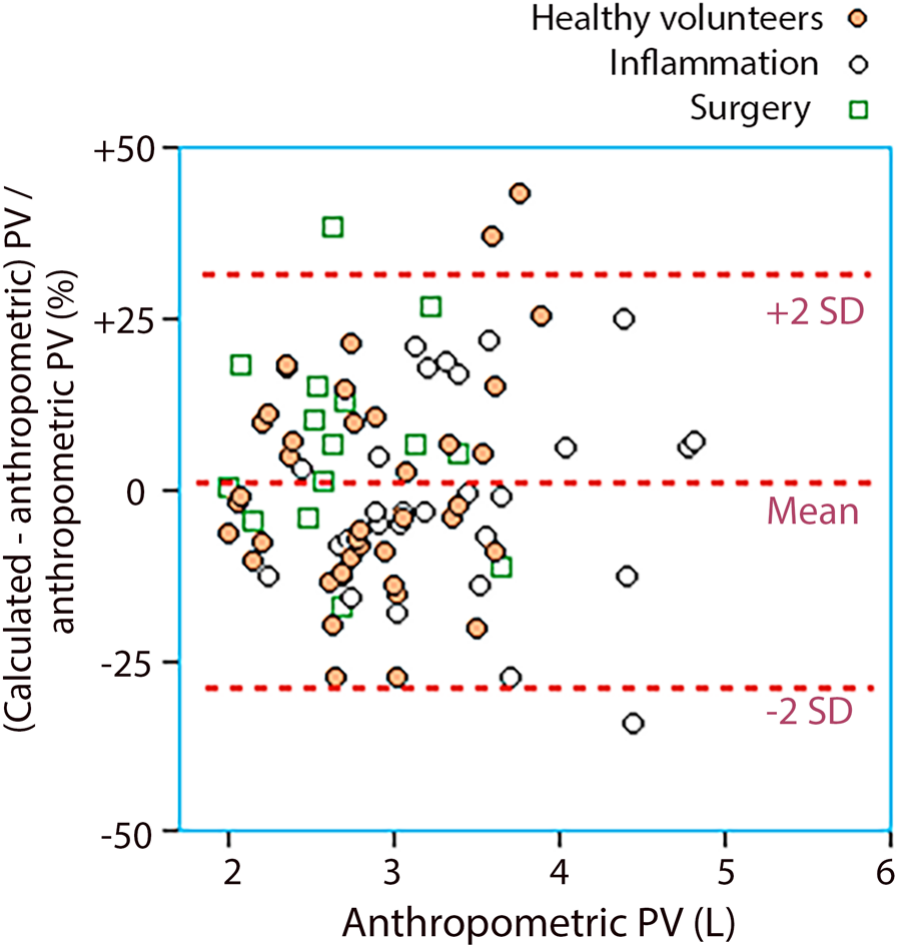
Relative deviation of the calculated PV from the anthropometric PV when the capillary leakage of albumin was corrected by manual extrapolation of the terminal elimination slope

The prediction error was lowest at 30–40 min and then averaged 0.31 ± 0.56 L when no correction for capillary leakage was applied, 0.04 ± 0.51 L when manual correction was used, and 0.13 ± 0.53 L when the kinetic correction was applied (Fig. 3). Hence, the albumin-based PV usually gave slightly higher values than the PV derived by anthropometry (Fig. 4).

The prediction error was no different for small and large PVs, but the SD was only half as large for PVs < 2.5 L as for PVs > 2.5 L. Moreover, the error was no different for the infusions with 5% albumin.

The prediction errors in the clinical groups are shown in Table 1**.** The prediction errors were also expressed in terms of the percent of the anthropometric value. These then became 9.8 ± 17.4, 1.0 ± 15.5%, and 3.4 ± 15.6% when the three different modes of handling the capillary leakage of albumin were applied. The relative error did not change for large PVs.

The deviation in accuracy in the “no correction” group could be handled by subtracting 0.3 L from all PV measurements, which then yielded an overall prediction error of 0.00 ± 0.56 L. In the subgroups, the prediction error then became +0.01 ± 0.52 L (volunteers), −0.14 ± 0.59 L (inflammation), and 0.29 ± 0.51 L (surgery).

## Simulation

**Fig. 5:**
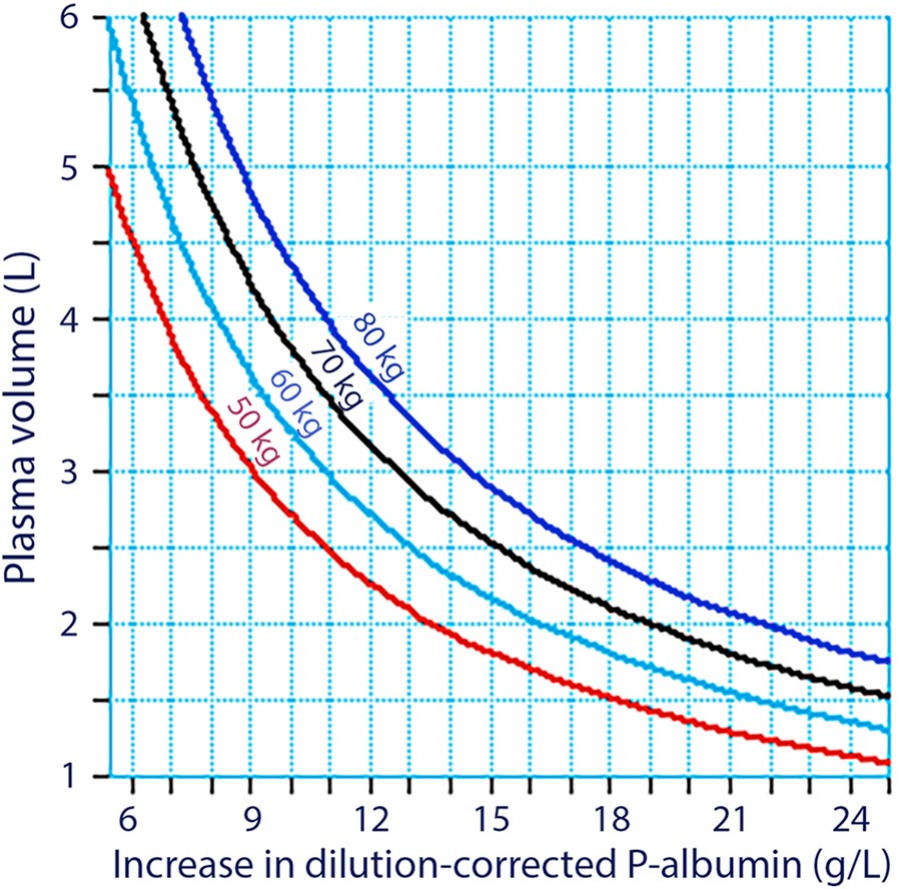
**A** Theoretical plasma volume for the difference between the plasma albumin concentration measured after and before infusing 3 mL/kg of 20% albumin over 30 min, with correction for plasma dilution of the post-infusion concentration [i.e., P-Alb_*t*_ (1 + DIL) minus P-Alb_*o*_]

Figure 5 shows the estimated PV for increasing differences between P-Alb_t_ (1 + DIL) and P-Alb_o_, i.e., the increase in P-Alb resulting from intravenous infusion of 3 mL/kg of 20% albumin after correction for dilution. Slightly displaced curves were obtained depending on body weight while the starting point, P-Alb_o_, did not matter. The calculations were made based on Eqs. 3 and 4 and do not involve the anthropometric estimates.

## Discussion

### Key results

Our analysis suggests a procedure that should make it possible to estimate the PV based on simple biochemical measurements when administrating 20% or even 5% albumin. The approach is based on comparing P-Alb and B–Hb measured before and after the albumin infusion. We explored the prerequisites for the methodology by comparing the output with PVs derived by anthropometric data. After infusing 3 mL/kg of 20% albumin over 30 min, the linearity analysis suggests that best chance to obtain a correct estimate of PV is to sample blood at the end of the infusion or 10 min later, while the accuracy will be is poorer both before and after these points.

Using the mean of these two timepoints, the highest correlation coefficient for all subjects was 0.80, which corresponds to a coefficient of determination (*r*^2^) of 0.64. The standard deviation of the PV estimates was relatively high (approximately 0.5 L), although the precision was much better when estimating small PVs than large ones (Fig. 3). The reason for this difference is apparent from Fig. 5**,** where hypovolemia is indicated by a greater fluid-induced increase in P-Alb than hypervolemia; this makes small measurement errors a less crucial issue.

### Correction for capillary leakage

One concern was that capillary leakage of albumin could distort the results. This was partially true, because variable capillary leakage rates probably explain why the linearity became worse over time. The PV equation appeared to be more reliable when the albumin dose was corrected for capillary leakage versus no correction. PV with kinetic correction worked slightly less well than the manual correction, which deviated by only 40 mL from the PV given by the anthropometric equations (Table 1). The reason why the kinetic correction offered no improvement is probably the pseudo-steady state of P-Alb and/or plasma dilution that frequently develops 1–2 h after infusion of 20% albumin; this had been noted in several of the underlying studies.

The accuracy of the prediction made without correction for capillary leakage can be improved by subtracting the average volume that differed between the PV derived by the anthropometric equations and the prediction based on our measurements of P-Alb and B–Hb. This difference averaged 0.3 L. Therefore, we believe that the most practical approach would be to use the uncorrected PV and simply subtract 0.3 L. This is especially relevant, because individual corrections for capillary leakage require serial blood sampling, which is difficult to perform clinically. One should note that the correction should differ slightly between the experimental situations, as shown in Table 1. By contrast, the precision of the predictions could not be markedly improved by corrections.

Our suggested correction of 0.3 L can be changed when a future study uses radioactive tracers to validate our method.

### Previous work

Margarson & Soni [3] pioneered an albumin-based estimation of PV by infusing 20% albumin in 12 septic patients before and after measuring PV and the capillary leakage using the radioactive iodine–albumin method. They infused 200 mL of 20% albumin over 90 s through a central venous catheter, a rate so high that it questions the clinical applicability of the method. The 95% limits of agreement between the two approaches were −660 mL to +440 mL, or from −16% to +11%, which is slightly narrower than we found; however, the average PV that was 1 L larger [4]. They did not extrapolate the isotope activity to zero time by linear regression based on the measurements performed during 30–40 min, as is conventional, but rather relied on the isotope activity measured at 10 min. Their albumin-estimated PV was 10–15% larger at 15 min than at 1 min, which is likely due to the combined effects of capillary leakage of albumin and a time delay which is due to that hyper-oncotic albumin recruits extravascular fluid via the lymphatic route [10].

Another study published at the same time included 70 patients with septic shock and 26 controls [5] but this study focused on oncotic-driven recruitment of extravascular fluid to the plasma and did not contain radioisotope measurements.

Other methods used to measure the PV do not rely on an increase of the intravascular albumin mass, as the present one does, but still apply the dilution principle to changes in P-Alb. The albumin molecules are first tagged with a foreign substance that can be measured by a specialized analysis equipment. These methods include ^131^I–iodine-labelled, Evan’s blue (a color dye), and indocyanine green (ICG) [1]. The ICG has a fast turnover, and the PV can be performed within 5 min. The elimination is zero during the first min as some time is required for the tracer to reach the liver, where the elimination occurs [11].

Alternatively, the red cell volume is measured by carbon monoxide, chromium or technetium, and then using the hematocrit to convert the result to the PV.

### The PV method in different clinical settings

We assumed that the usefulness of the albumin-based PV would be limited in the clinical settings we studied as compared to the data obtained in volunteers. However, the between-group differences were not dramatic and the precision was even quite similar when we tested the equations in patients with inflammation and in patients undergoing surgery. The 15 post-burn patients had clearly increased body weights compared with their pre-burn weights—this is a remnant of the aggressive initial fluid resuscitation. They were weighed just before the experiment, and therefore, they received albumin in proportion to their actual (increased) body weight. The well-maintained linearity implies that fluid overloading increases both the body weight and the PV to a similar degree, allowing the anthropometric equations to account for them.

In contrast, the decrease in arterial pressure during induction of anesthesia in the surgery group re-distributes interstitial fluid to the plasma [12], which is likely to create a predictable positive difference between the albumin-estimated and the anthropometric PV regardless of when the body weight is measured. This is also what happened, although the clinician can subtract the mean deviation from the PV here, too, according to the value shown in Table 1**.**

All our subjects were in a good hemodynamic situation and were judged to have a stable PV when the experiments were performed, although the clinical value of the method would be greatest when disclosing PVs that deviate greatly from the expected values. Hence, we may assume that patients with hemorrhage, dehydration, or fluid overload would show deviating PV results relative to the anthropometric equations. At present, we cannot judge the accuracy of the presented method in severely sick patients. Theoretically, the method would work well in all scenarios where tracer methods can be applied, i.e., in patients with an intact circulation and not during hemorrhage.

The slopes in Fig. 5 predict that the method will be more precise in hypovolemic than in hypervolemic states, which we illustrate with worked-through example. A clinician decides to provide 3 mL/kg of 20% albumin on the suspicion of hypovolemia in a patient weighing 70 kg. The infusion-associated increase in the dilution-corrected P-Alb concentration of 19 g/L indicates that the PV was 2 L when the infusion started, which confirms the diagnosis. In addition, the uncorrected increase in P-Alb corresponded to the PV after infusion ends. However, the rise would be 13 g/L if the patient was normovolemic when the infusion started (PV = 3 L) and only between 9 and 10 g/L if the patient was hypervolemic (PV = 4 L). Hence, the change in P-Alb would be twice as large in the hypovolemic setting compared to the hypervolemic situation.

### Limitations

The purpose of the present study was to explore the feasibility of a proposed setup for estimating the PV during albumin therapy. We did not validate the method by isotopes at this stage, because ethical concerns arise if the setup is uncertain. Therefore, the “accuracy” in the present study is not a fully adequate term; the albumin-estimated PVs were compared to the mean of two anthropometric equations, albeit these equations were based on tracer techniques. Hence, the control values were not obtained in the same individuals as those to whom we administered albumin. The control values always refer to a stable volunteer situation. These issues are severe limitations. Another limitation is that data were obtained from several separate studies, albeit the same protocol was used in all of them.

An infusion of 3 mL/kg of 20% albumin expands the PV by twice the infused volume which, in this study, equals to approximately 400 mL. This PV expansion may not be tolerated in patients with heart failure, volume overload, or other debilitating conditions. However, the method is suggested to be applied when PV expansion is indicated for clinical purposes. Hence, we do not suggest that 200 mL of 20% albumin should be infused exclusively to measure the PV. Therefore, the contraindications for using our method are the same as the contraindications for using 20% albumin.

There was no clinical indication for albumin treatment in the volunteers in this study, while the indication was relative in the other two groups. A clear indication for albumin treatment might be considered to be at hand in critically ill and/or hypovolemic patients, but such patients were not included in this study.

Novel contributions in this article are that we identify the timepoints when the PV is most likely to be accurately measured, we compare the effects of two corrections for capillary leakage versus no correction, presenting adequate equations for this purpose, and the insight that Hb correction measures the PV at baseline while leaving out the Hb correction gives the PV expansion at the end of the infusion.

The strengths of the study include our use of a setup that is feasible to apply clinically and hereby proven to work well in several settings. A standardized protocol was used for all experiments. We followed the volunteers and patients for several hours and identified the optimal time for the blood sampling. We also demonstrated that the precision of the method is unlikely to differ greatly across different on clinical settings.

## Conclusions

We estimated the PV in 86 subjects based on measurement of plasma albumin and blood hemoglobin before and shortly after infusing approximately 200 mL of 20% albumin over 30 min. The calculations used the dilution principle, which means that the PV equals the amount of added albumin divided by the plasma albumin concentration. Our results demonstrate a reasonably good correlation between the albumin method and the PV as obtained by two anthropometric methods (correlation coefficient 0.80). A confirmatory study with isotopes is needed and our results suggest how to set up such a study. We find that the method is feasible to apply clinically and, after further evaluation, can be used to estimate the PV whenever 20% albumin is provided for clinical purposes.

## Supplementary Information

Additional file 1.

## Data Availability

All individual data are given in Supplementary Material 1.

## Abbreviations

B–Hb: Blood–hemoglobin
BV: Blood volume
BW: Body weight
DIL: Plasma dilution based on blood hemoglobin
H: Height
Hct: Hematocrit
SD: Standard deviation
P-alb: Plasma albumin
ΔP-alb: Change in plasma albumin
